# Erlotinib and Onalespib Lactate Focused on EGFR Exon 20 Insertion Non-Small Cell Lung Cancer (NSCLC): A California Cancer Consortium Phase I/II Trial (NCI 9878)

**DOI:** 10.1016/j.cllc.2021.05.001

**Published:** 2021-05-15

**Authors:** Jonathan W Riess, Karen L Reckamp, Paul Frankel, Jeffrey Longmate, Karen A Kelly, David R Gandara, Caroline M Weipert, Victoria M Raymond, Harold N Keer, Philip C Mack, Edward M Newman, Primo N Lara

**Affiliations:** a Division of Hematology/Oncology, Department of Internal Medicine, UC Davis Comprehensive Cancer Center, University of California, Davis, Sacramento, CA; b City of Hope Comprehensive Cancer Center, Duarte, CA; c Guardant Health, Redwood City, CA; d Tisch Cancer Institute–Mount Sinai, New York, NY; e Astex Pharmaceuticals, Pleasanton, CA; f Division of Medical Oncology, Department of Medicine, Cedars-Sinai Medical Center, Los Angeles, CA

**Keywords:** Heat shock protein inhibitors, circulating tumor DNA, EGFR Exon 20 insertion, Heat shock protein 90, EGFR tyrosine kinase inhibitor

## Abstract

This study examined the safety and tolerability of erlotinib and the heat shock protein 90 inhibitor onalespib in *EGFR*-mutant non–small cell lung cancer (NSCLC). The phase II component examined preliminary efficacy in epidermal growth factor receptor exon 20 insertion (EGFRex20ins) NSCLC. Overlapping toxicities, mainly diarrhea, limited the tolerability of the combination. EGFRex20ins circulating tumor DNA (ctDNA) was detected in the majority of patients; failure to clear ctDNA was consistent with lack of tumor response.

**Background:**

Onalespib is a novel heat shock protein 90 inhibitor (HSP90i). Previous preclinical and clinical studies with HSP90i have demonstrated activity in *EGFR*-mutant non–small cell lung cancer (NSCLC). This study sought to determine the safety and tolerability of onalespib plus erlotinib in *EGFR*-mutant NSCLC and to evaluate the preliminary efficacy of the combination in epidermal growth factor receptor exon 20 insertion (*EGFR*ex20ins) NSCLC.

**Patients and Methods:**

Standard 3+3 dose escalation was followed by a phase II expansion in *EGFR*ex20ins. The phase II component targeted a response rate of 25% versus a background rate of 5%. Prospective next-generation sequencing (NGS) of 70 cancer-related genes, including *EGFR*, via plasma circulating tumor DNA (ctDNA) was performed. Toxicity was graded by Common Terminology Criteria for Adverse Events (CTCAE), version 4, and response was determined by Response Evaluation Criteria in Solid Tumours (RECIST) 1.1.

**Results:**

Eleven patients were treated (nine dose escalation, two dose expansion). Two dose-limiting toxicities (DLTs) occurred in dose level (DL) 0 and zero in DL −1 (minus). In 10 *EGFR*ex20ins patients, no responses were observed, median progression-free survival was 5.4 months (95% confidence interval, 0.9–5.7), and the disease control rate (DCR) was 40% (median, 3.5 months). *EGFR*ex20ins was detected in nine of 10 ctDNA samples at baseline; on-treatment ctDNA clearance was not observed. Grade 3 diarrhea was the predominant toxicity in 45% of patients. The recommended phase II dose is DL −1 (minus): erlotinib 150 mg orally every morning and onalespib 120 mg/m^2^ intravenously on days 1, 8, and 15 every 28 days.

**Conclusion:**

Overlapping toxicities of erlotinib and onalespib, mainly diarrhea, limited the tolerability of this combination, and limited clinical activity was observed, so the trial was closed early. Plasma *EGFR*ex20ins ctDNA was detected in the majority of patients; failure to clear ctDNA was consistent with lack of tumor response (NCT02535338).

## Introduction

Epidermal growth factor receptor exon 19 deletion (*EGFR* E19del) and the *EGFR* L858R mutation in exon 21 represent the vast majority of *EGFR*-activating mutations and are exquisitely sensitive to approved EGFR tyrosine kinase inhibitors (TKIs). Erlotinib is a first-generation EGFR TKI and one of several EGFR TKIs currently FDA approved for these canonical *EGFR* mutations based on improved response rates and progression-free survival (PFS) compared with platinum-based chemotherapy.^1^ *EGFR* exon 20 insertions (*EGFR*ex20ins) are a collection of driver mutations comprising approximately 4% to 12% of all *EGFR* mutant lung cancers.^2,3^ Although *EGFR*ex20ins usually have the same transforming ability as more common *EGFR*-activating mutations and are thus considered oncogenic drivers, they are typically unresponsive to first- and second-generation EGFR TKIs due to the modified structures of their kinase domains.^4^ Osimertinib, although FDA approved for *EGFR* E19del, L858R, and T790M-positive NSCLC, is not approved for *EGFR*ex20ins, but it has modest preclinical activity and anecdotal reports of response and is being studied in a National Cancer Institute (NCI) cooperative group study (NCT03191149).^5,6^ Newer generation EGFR TKIs, such as poziotinib and mobocertinib (TAK-788) or the EGFR/mesenchymal–epithelial transition (MET) bifunctional antibody amivantamab (JNJ-372), have improved clinical activity with higher response rates and are in clinical trials but are not yet approved for routine clinical use.^7–10^

Heat shock protein (HSP) complexes act as molecular chaperones that participate in stabilizing and activating client proteins. Cancer cells use HSP90 to protect mutated and overexpressed proteins from ubiquitin-mediated degradation.^11^ *EGFR*ex20ins mutations have demonstrated interaction with and dependence on HSP90 in vitro.^12,13^ In patients harboring *EGFR*ex20ins, modest activity was observed with the HSP90 inhibitor luminespib (AUY922) as a single agent with an overall response rate of 17% and median PFS of 2.9 months (95% confidence interval [CI], 1.4–5.6); a high rate of diarrhea and an unusual side effect of ocular visual changes were observed.^14^

Onalespib lactate (AT13387) is a non-ansamycin small molecule that potently inhibits HSP90 chaperone activity and promotes degradation of client oncoproteins that mediate resistance to EGFR TKIs.^15,16^ Maintaining prolonged knockdown of HSP90 client proteins has been a challenge in the development of this class of drugs in NSCLC. Oncoprotein knockdown by onalespib is prolonged relative to its plasma half-life in NSCLC cell lines and xenotransplants.^17^ Preclinical activity of HSP90 inhibition with onalespib has been shown in several oncogene-driven tumors, including EGFR TKI-sensitive and -resistant *EGFR*-mutant NSCLC, anaplastic lymphoma kinase–rearranged NSCLC, *BRAF*-mutant melanoma both sensitive and resistant to dual BRAF and MEK inhibition, and *KIT* mutated gastrointestinal stromal tumors both sensitive and resistant to imatinib.^18–20^ Onalespib has single-agent activity in *EGFR*-mutant NSCLC, is synergistic with erlotinib in *EGFR*-mutant NSCLC, and delays the emergence of resistance to EGFR TKI treatment in cell lines and xenotransplants.^20^

In addition to determining the safety and efficacy of erlotinib plus onalespib in a phase I dose escalation trial in *EGFR*-mutant NSCLC, the phase II portion of this study sought to explore whether combination strategies with HSP90 inhibition and EGFR TKIs may increase activity specifically in *EGFR*ex20ins NSCLC.

## Methods

### Eligibility Criteria

Eligible patients were required to have NSCLC harboring an *EGFR*-activating mutation. In dose escalation, patients with *EGFR* E19del, L858R, L861Q, or G719X must have received prior EGFR TKI therapy for metastatic disease with evidence of disease progression. Patients with *EGFR*ex20ins must have received prior platinum-based chemotherapy with disease progression on or after treatment. Patients must have had an Eastern Cooperative Oncology Group performance status ≤ 2; measurable disease according to Response Evaluation Criteria in Solid Tumors (RECIST) version 1.1; and adequate hematologic, renal, and liver function. Key exclusion criteria included active central nervous system metastasis (treated central nervous system metastasis allowed); corrected QT interval by Fredericia (QTcF) *>* 470 ms; left ventricular ejection fraction ≤ 50% as demonstrated by echocardiogram or multigated acquisition (MUGA) scan; or any major medical condition that would interfere with participation.

The study was approved by independent ethics review boards and in accord with an assurance filed with and approved by the Department of Health and Human Services by each site (NCT02535338). The study was conducted according to the tenets of the Declaration of Helsinki. The study was approved by the Central Institutional Review Board for the NCI in accordance with an assurance filed with and approved by the Department of Health and Human Services at each of the participating investigational centers. All patients provided written informed consent prior to study participation.

### Study Design and Treatment

**Figure 1:**
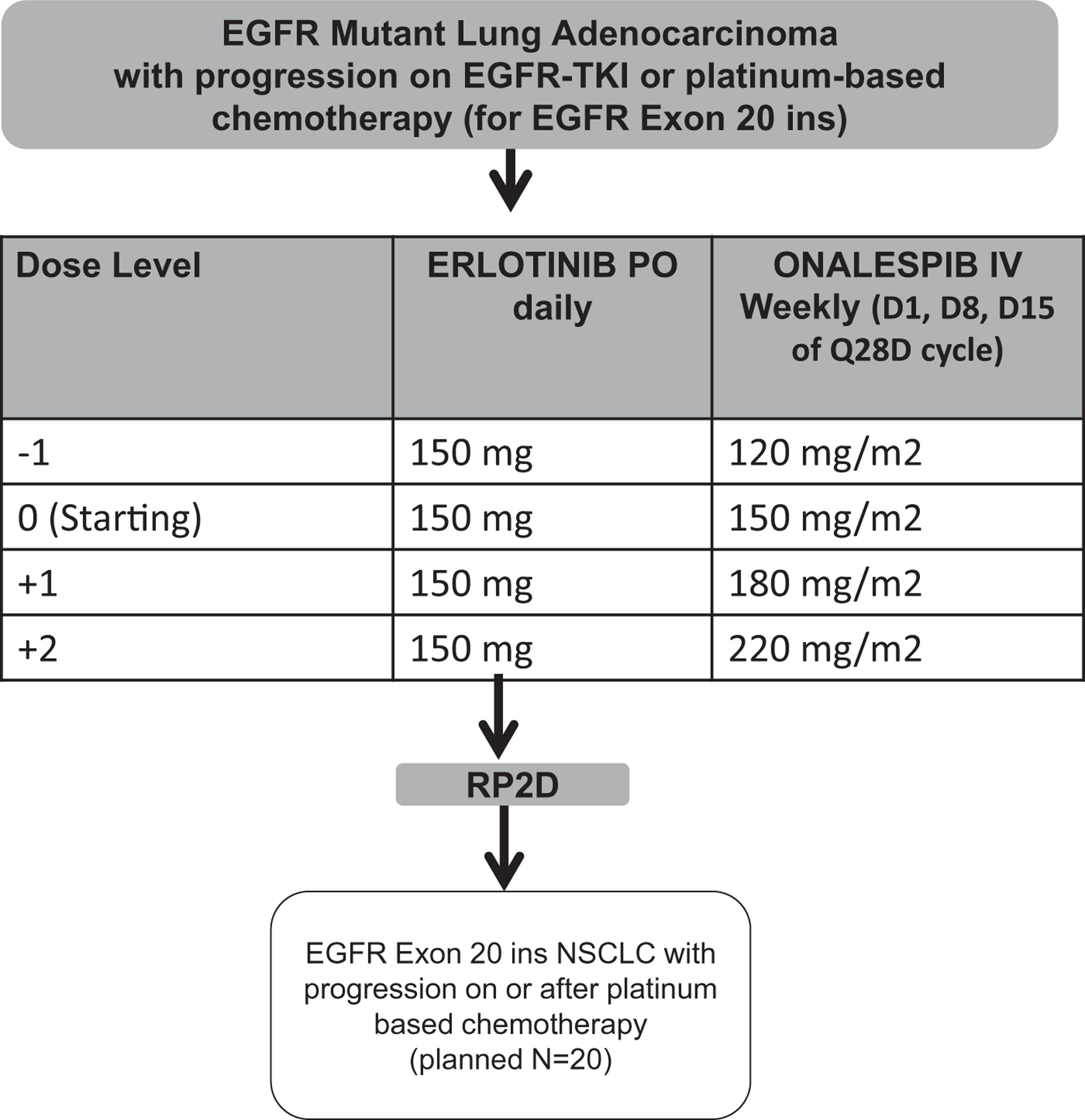
Diagram of Dose Escalation and Expansion Cohort. Abbreviations: EGFR = epidermal growth factor receptor; TKI = tyrosine kinase inhibitor.

The phase I portion, an open label, 3+3 dose escalation, was designed with four dose levels. Erlotinib was administered orally at 150 mg daily at all dose levels, and no more than two dose reductions (100 mg and 50 mg) were permitted for drug-related adverse events. Onalespib was administered intravenously (I.V.) on days 1, 8, and 15 on a cycle of every 28 days. The starting dose of onalespib in combination with erlotinib (DL0) was 150 mg/m^2^ I.V. with two planned dose escalation levels (180 mg/m^2^ and 220 mg/m^2^) and a dose de-escalation level of onalespib at 120 mg/m^2^ (DL −1 (minus)) if more than one dose-limiting toxicity (DLT) was observed at the starting dose level (Figure 1). Treatment was continued until disease progression, occurrence of unacceptable adverse events, investigator determination, or patient withdrawal from the study. Specific additional discontinuation criteria included patients with interstitial lung disease of any grade related to the study drugs, recurrent grade (Gr) 3 diarrhea despite medical management, and recurrent Gr 3 rash despite medical management.

Adverse events were graded by Common Terminology Criteria for Adverse Events (CTCAE), version 4. DLTs were defined as ≥Gr 3 non-hematologic toxicity except nausea, vomiting, or diarrhea that could be controlled by appropriate medical intervention or prophylaxis and that resolved to ≤Gr 1 within 48 hours with medical intervention, except electrolyte toxicities that can be corrected to ≤Gr 1 within 48 hours. Gr 3 rash attributed to the combination was considered a DLT if it remained Gr 3 despite maximal medical management (including oral antibiotic and topical or oral steroids) for *>*72 hours. Hematologic toxicities qualifying as DLTs included febrile neutropenia; Gr 4 neutropenia for *>*7 days or thrombocytopenia *<* 25,000/mm^3^ (Gr 4) if associated with a bleeding event that did not result in hemodynamic instability but required an elective platelet transfusion; or a life-threatening bleeding event that resulted in urgent intervention and admission to an intensive care unit. Delay in starting the second cycle of ≥14 days due to toxicity related to one or more protocol drugs was also considered a DLT. The first 28-day cycle was considered the DLT period.

For the dose-expansion phase II portion of the study, patients with *EGFR*ex20ins were treated at the recommended phase II dose (RP2D) of erlotinib and onalespib determined in the dose-escalation portion of the study (Figure 1). This cohort enrolled patients with metastatic or recurrent NSCLC who harbored an *EGFR*ex20ins and had progressive disease on platinum-based chemotherapy. The primary endpoint for the phase II segment was best objective response by RECIST 1.1 criteria and governed by Simon’s two-stage minimax design, implemented to distinguish a 25% response rate from an assumed background rate of 5%, with 10% type I and type II error rates. Time on treatment was defined as time from treatment initiation to discontinuation for any reason. Disease control was defined as stable disease or better on at least two assessments approximately 8 weeks apart. The planned initial stage was to enroll 13 subjects, continuing if one or more responded. The target planned total accrual for this phase II expansion cohort was 20 subjects, with three responses considered encouraging for further development in the relevant class of patients.

### EGFR *Mutation Analysis*

*EGFR* mutational analysis was per local Clinical Laboratory Improvement Amendments (CLIA) regulations as part of standard of care. Subjects were permitted to enroll if the *EGFR* mutation was identified based on tissue mutational analysis or circulating tumor DNA (ctDNA) mutational analysis.

### Pharmacokinetic Analysis

Plasma was collected for pharmacokinetic analysis of onalespib. Blood samples at C1D8 were drawn into lithium heparin–containing tubes prior to onalespib infusion and at 0.5, 1, 2, 3, 4, 6, 8, and 25 hours after the start of onalespib infusion.

### Plasma Mutation Analysis

ctDNA analysis was completed using a commercially available targeted next-generation sequencing (NGS) assay (Guardant360; Guardant Health, Redwood City, CA) that is CLIA certified, College of American Pathologists accredited, and New York State Department of Health approved. ctDNA isolation and analytic methods have been described previously.^21,22^ Briefly, two 10-mL tubes of whole blood were collected in Cell-Free DNA BCT blood collection tubes (Streck, Inc., La Vista, NE) prior to treatment, every two cycles (every 8 weeks), and at progression. At the time of this study, Guardant360 included targeted analysis of single-nucleotide variants in 68 to 70 clinically relevant cancer genes, as well as small insertions/deletions, gene fusions, and copy number gains (CNGs) in a subset of genes including *EGFR* and common resistance mechanisms to EGFR TKIs (including *EGFR* CNG, *MET* CNG, *EGFR* T790M, oncogenic fusions, *BRAF*, and *HER2* CNG). The variant allele fraction (VAF) was calculated by dividing the number of tumor-derived DNA molecules at each location by the total number of unique ctDNA molecules at the given nucleotide position. We used 5% VAF-detectable *EGFR*ex20ins ctDNA at baseline to analyze clinical outcomes based on a previous association of 5% ctDNA or above with overall survival in NSCLC.^23^

## Results

Eleven patients were treated: nine in dose escalation and two in expansion; the patient characteristics are provided in Table 1. The majority of patients (10/11) had lung cancers harboring *EGFR*ex20ins and were heavily pretreated; over half of the patients (54%) received three or more prior lines of therapy. Mean systemic area under the curve exposures overlapped for the onalespib dose levels of 120 mg/m^2^ and 150 mg/m^2^ due to higher variability at the lower dose (see Supplemental Table 1 in the online version at doi:10.1016/j.cllc.2021.05.001).

Two DLTs (Gr 3 maculopapular rash and Gr 3 hypophos-phatemia) occurred among the three patients treated at dose level 0 (erlotinib 150 mg PO daily, onalespib 150 mg/m^2^ IV D1, D8, D15 on a 28-day cycle), so patients were subsequently enrolled in DL −1 (minus), per the 3+3 design. No DLTs occurred at DL −1 (minus) (erlotinib 150 mg PO daily, onalespib 120 mg/m^2^ IV D1, D8, D15 on a 28-day cycle). The Gr 3 maculopapular rash at DL0 was asymptomatic but involved more than 30% of body surface area. The Gr 3 hypophosphatemia met DLT criteria, as it was not corrected to grade 1 or better within 48 hours. Common drug-related adverse events (AEs) of any grade were diarrhea (100%), rash (64%), fatigue (55%), increased bilirubin (27%), and nausea/vomiting (55%). The most common drug-related Gr 3 AEs were diarrhea (45%), maculopapular rash (9%), and hypophosphatemia (9%) (Table 2).

**Figure 2:**
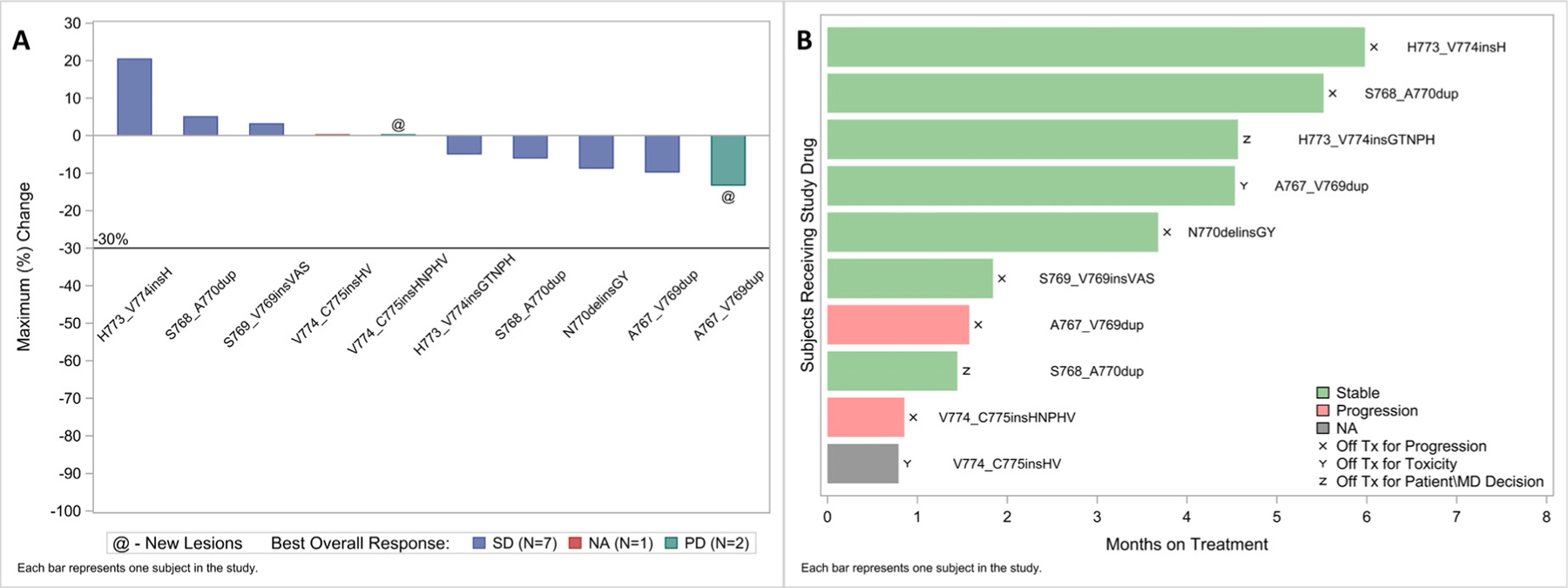
A, Waterfall Plot of Best Response and B, Months on Treatment Among *EGFR*ex20ins NSCLC. Abbreviation: *EGFR*ex20ins = *EGFR* exon 20 insertion.

**Figure 3:**
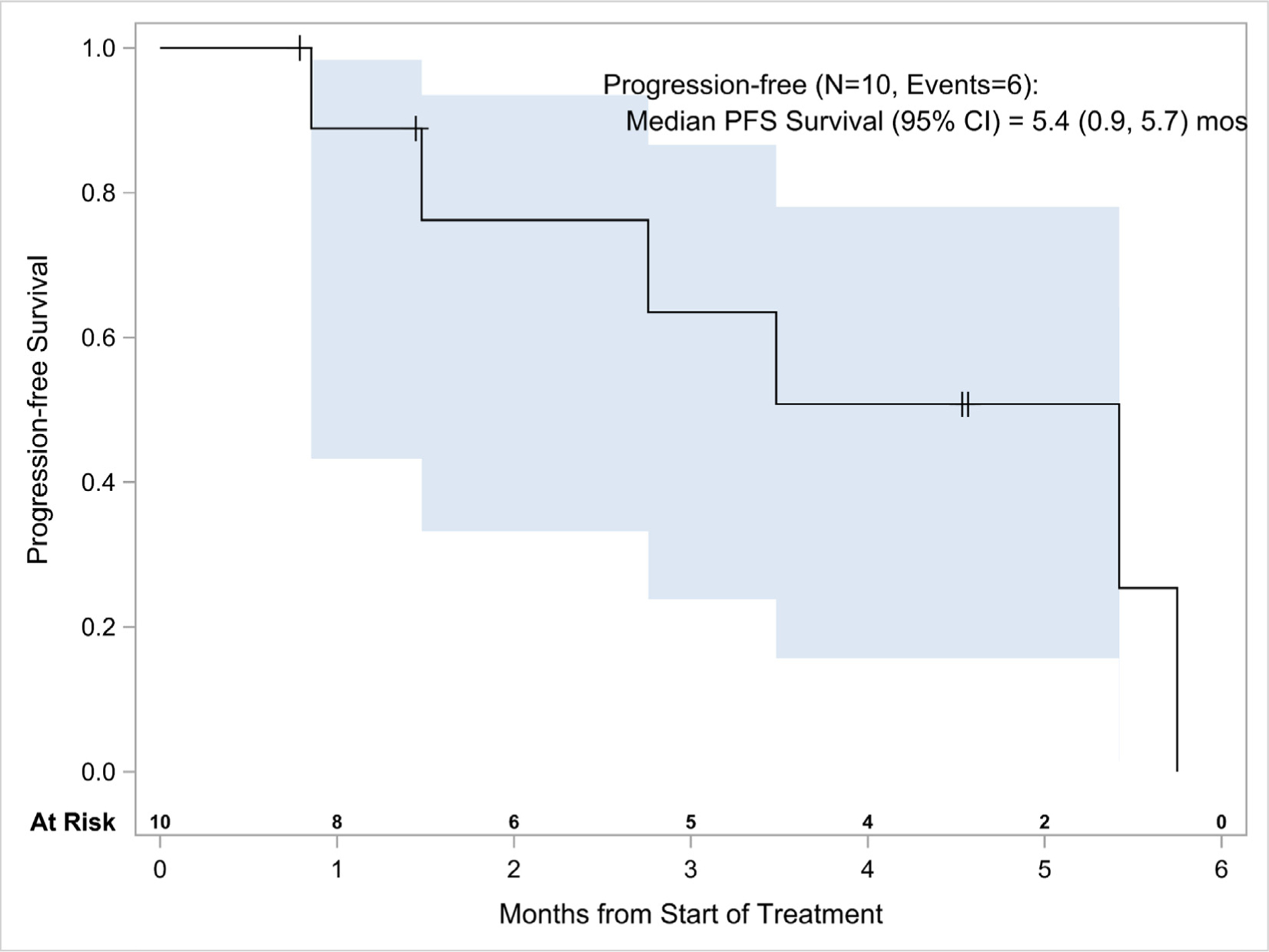
PFS in *EGFR*ex20ins Patients Treated with Erlotinib and Onalespib. Abbreviations: *EGFR*ex20ins = *EGFR* exon 20 insertion; PFS = progression-free survival.

No objective responses were noted in the 11 patients treated with erlotinib and onalespib (10 *EGFR*ex20ins and one *EGFR* E19del), although half of the patients (5/10) with *EGFR*ex20ins treated with erlotinib and onalespib had some degree of tumor shrinkage (Figure 2). In patients with *EGFR*ex20ins, the median number of cycles administered was two (range, 1–6), with two patients discontinuing for toxicity with a median disease control of 3.5 months (range, 1.3–6.6) and a disease control rate of 40%. Median PFS was 5.4 months (95% CI, 0.9–5.7) (Figure 3). Of the 10 patients with *EGFR*ex20ins, six were removed from treatment for progressive disease, two for toxicity (persistent Gr 2 diarrhea), and two by investigator decision.

*EGFR*ex20ins was detected in ctDNA at baseline in nine of 10 patients and failed to clear (ie, alterations remaining detectable) in all nine patients with longitudinally collected samples after initiation of treatment. Among the 10 patients treated, eight unique *EGFR*ex20ins were observed that represent the most frequent *EGFR*ex20ins encountered in clinical practice, including the two most common *EGFR*ex20ins: A797_V769dup and S768_A770dup.^3^ Comutations were assessed, and of the nine patients with detectable *EGFR*ex20ins ctDNA, seven patients (67%) at baseline also harbored p53 co-mutations (78% at end of therapy). Supplemental Figure 1 (see the online version at doi:10.1016/j.cllc.2021.05.001) represents co-alterations at baseline and at the end of treatment. In the absence of objective responses, it is unclear that any changes in co-alterations at the end of treatment represented potential acquired resistance mechanisms versus passive acquired alterations^33,34^.

**Figure 4:**
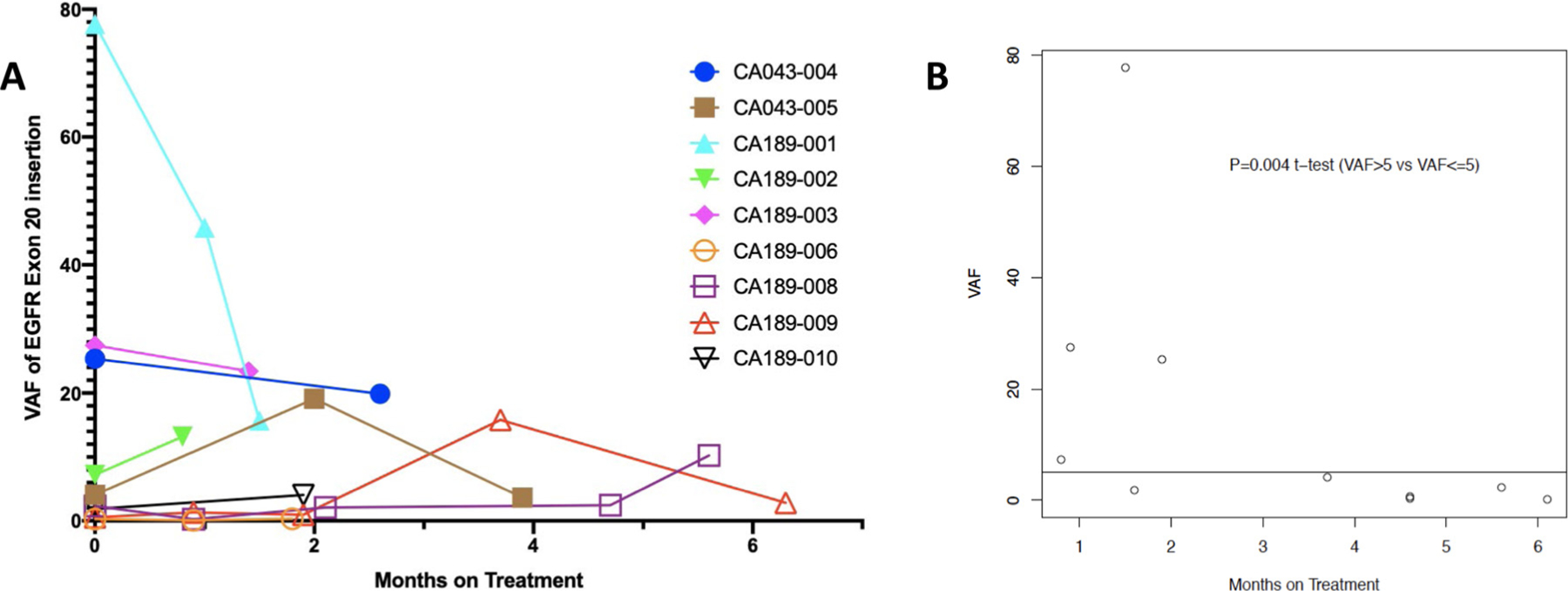
A, Dynamics of *EGFR*ex20ins VAF From Plasma ctDNA. Nine of 10 Patients With *EGFR*ex20ins Were Detected at Baseline. No Patients With Detectable *EGFR*ex20ins ctDNA at Baseline Cleared the Mutation During Treatment, Corresponding With the Lack of Clinical or Radiographic Response. B, Baseline VAF and Months on Treatment. Cutpoint of 5% *EGFR*ex20ins VAF Was Prognostic for Time on Treatment. Abbreviations: ctDNA = circulating tumor DNA; *EGFR*ex20ins = *EGFR* exon 20 insertion; VAF = variant allele fraction

*EGFR*ex20ins baseline VAF was associated with time on treatment. With *EGFR*ex20ins low VAF (*<*5%), mean time on treatment was 4.37 months; with high VAF (≥5%), the mean time on treatment was 1.28 months (*P* = .004). The overall impact of treatment on VAFs of the *EGFR*ex20ins was modest (Figure 4). Also included was one patient with a non-exon 20 insertion who was previously treated with several lines of therapy and whose tumor harbored *EGFR* E19del/T790M alterations and the hotspot *PIK3CA* mutation R88Q at baseline (after progression on multiple lines of chemotherapy, including erlotinib, capmatinib, afatinib, osimertinib, and necitumumab). This patient had progression of disease at first response assessment with concomitant failure to clear ctDNA.

## Discussion

HSP90 inhibitors have shown modest clinical activity in *EGFR*-mutant lung cancer, including in combination with erlotinib, and modest activity as a single agent in *EGFR*ex20ins NSCLC.^14,24^ In this study, the addition of onalespib to erlotinib in *EGFR*-mutant NSCLC among 10 of 11 patients harboring *EGFR*ex20ins yielded no objective responses in the first 11 patients treated, including eight patients dosed at the RP2D (onalespib at 120 mg/m^2^ IV on days 1, 8, and 15 and erlotinib at 150 mg PO every day on a 28-day cycle). Given the lack of response and the changing landscape of treatment of advanced *EGFR*ex20ins with the development of next-generation EGFR inhibitors such as poziotinib, mobocertinib, and amivantamab, the investigators recommended early trial closure prior to accrual completion following consultation with the trial sponsor (NCI).

Overlapping toxicities of erlotinib and onalespib, mainly diarrhea and other gastrointestinal side effects, limited the tolerability of this combination. At the first dose level, DLTs in two of three patients (rash and hypophosphatemia) necessitated a dose de-escalation, with onalespib at 120 mg/m^2^ I.V. on days 1, 8, and 15 IV and erlotinib at 150 mg orally every day on a 28-day cycle determined to be the maximum tolerated dose (MTD) and RP2D.

Pharmacokinetic (PK) studies were evaluated with onalespib in the nine subjects in dose escalation (see Supplemental Table 1 in the online version at doi:10.1016/j.cllc.2021.05.001). The PK profile was similar to what has been reported previously.^25^ However, doses achieved in combination with erlotinib were limited by overlapping toxicity and were lower than in the phase I trial of single-agent onalespib. In the single-agent study, the MTD of weekly onalespib was 260 mg/m^2^ on days 1, 8, and 15 of a 28-day cycle and 120 mg/m^2^ twice weekly on a 28-day cycle.^25^ Lower doses of onalespib may limit target engagement. Although onalespib has been shown to concentrate in tumors in preclinical in vivo models, with a half-life up to 65 hours which far exceeds the plasma half-life, suppression of mutant *EGFR* in patients may be more transient.^15,17^

*EGFR*ex20ins are heterogeneous, with over 60 different mutations noted by NGS in the largest published series.^3^ NGS comprehensively assesses the spectrum of possible *EGFR*ex20ins for clinical decision making. To our knowledge, this is the first prospective, therapeutic clinical trial demonstrating the feasibility of detecting and monitoring ctDNA in a prospective therapeutic trial for *EGFR*ex20ins in NSCLC. This study demonstrated that a well-validated, commercially available targeted NGS ctDNA assay (Guardant360) was able to reliably detect *EGFR*ex20ins in 90% of patients (9/10) at baseline, often following several lines of previous therapy.

Lack of objective radiographic response corresponded with the failure to clear *EGFR*ex20ins from ctDNA following treatment. Previous studies have shown that the VAFs of targetable alterations tends to decrease following effective therapy. Moreover, clearance of these alterations from ctDNA has been associated with response to therapy and improved outcomes in numerous cancer types, including NSCLC with common *EGFR*-activating mutations E19del and L858R.^26,27^ In this study, the failure to reach undetectable levels of ctDNA in any patient following treatment corresponded to a lack of clinical or radiographic response to erlotinib and onalespib. This prospective trial adds further evidence to support the use of ctDNA to monitor treatment response (or lack thereof) and may aid in the early identification of patients who may derive more or less benefit from specific *EGFR*ex20ins-directed therapeutics in future clinical trials. *TP53* co-mutations at baseline were detected in the majority of patient ctDNA samples (6/9, 67%), comparable to the rate of p53 co-mutation in the largest known series of *EGFR*ex20ins with broad genomic profiling in tissue (56%).^3^ *TP53* co-mutations have also been shown to be prognostic for inferior clinical outcomes, including overall survival, in NSCLC harboring the most common *EGFR* mutations, E19del and L858R, and potentially a negative predictive factor for clinical outcomes to EGFR TKIs in these less common *EGFR* mutations, as well.^28^ Future studies should also examine the impact of p53 mutations on clinical outcomes in less common *EGFR* mutations such as *EGFR*ex20ins NSCLC.

Interestingly, patients with low or undetectable *EGFR*ex20ins VAFs at baseline (defined as *<*5%) had the longest time on treatment compared with high baseline VAFs (≥5%; mean time on treatment, 4.37 months vs. 1.28 months), with the majority of these patients being removed from the study for progressive disease (Figure 4). Similar to what was observed in a previous study with a comparable cutpoint dichotomizing patients with a VAF of *<*5% versus ≥5%, our study found that patients with a VAF of *<*5% had improved outcomes.^23,29,30^ Although further validation of ctDNA levels as a potential prognostic biomarker is necessary, improved clinical outcomes with lower VAFs may be due to several factors, including performance status, extent of disease burden, a more aggressive tumor phenotype, or fewer co-occurring alterations observed in the sample.

In a single-agent study of the HSP90 inhibitor luminespib (AUY922), overall response rate was 17%, and median PFS was 2.9 months (95% CI, 1.4–5.6). Although no responses were observed after adding the EGFR TKI erlotinib to the HSP90 inhibitor onalespib, median PFS was comparable at 5.4 months (95% CI, 0.9–5.7). Preclinical activity of other HSP90 inhibitors has been shown in combination with next-generation EGFR TKIs, such as osimertinib, that have more activity against *EGFR*ex20ins NSCLC than erlotinib.^31,32^ With next-generation EGFRex20ins inhibitors in clinical development, both TKIs and monoclonal antibodies, the addition of HSP90 inhibition may provide enhanced clinical activity, although the tolerability of these combinations may also be limited by gastrointestinal side effects such as diarrhea and other overlapping toxicities.^7^

### Clinical Practice Points

- Onalespib is a novel heat shock protein 90 inhibitor (HSP90i). Previous preclinical and clinical studies with HSP90i have demonstrated activity in *EGFR*-mutant non–small cell lung cancer (NSCLC).
- This study sought to determine the safety and tolerability of onalespib plus erlotinib in *EGFR*-mutant NSCLC and evaluate the preliminary efficacy of the combination in *EGFR* exon 20 insertion (*EGFR*ex20ins) NSCLC.
- The recommended phase II dose is erlotinib 150 mg orally every day and onalespib 120 mg/m^2^ intravenously weekly (on days 1, 8, and 15 of a 28-day cycle).
- Overlapping toxicities of erlotinib and onalespib limited the tolerability of this combination, with grade 3 diarrhea being the predominant toxicity in 45% of patients, and the combination demonstrated limited activity in *EGFR*ex20ins NSCLC, as no responses were observed.
- Plasma *EGFR*ex20ins circulating tumor DNA (ctDNA) was detected in the majority of patients; failure to clear ctDNA was consistent with lack of tumor response

## Supplementary Material

1

## Figures and Tables

**Table 1:** Patient Demographics and Clinical Characteristics

Patient Characteristics (*N* = 11)	
Age (y), median (range)	60 (51–70)
Gender, *n* (%)	
Male	3 (27)
Female	8 (73)
ECOG performance status, *n* (%)	
0	4 (36)
1	6 (55)
2	1 (9)
Histology, *n* (%)	
Adenocarcinoma	9 (82)
Adenosquamous carcinoma	1 (9)
Non–small cell carcinoma - NOS	1 (9)
*EGFR*ex20ins, *n* (%)	
Yes	10 (91)
No (EGFR E19del/PIK3CA R88Q)	1 (9)
Prior treatments (1; 2; ≥3), *n* (%)	1 (9); 4 (36); 6 (54)
*EGFR*ex20ins treated with prior EGFR TKI, *n* (%)	5 of 10 (50)

Abbreviations: ECOG = Eastern Cooperative Oncology Group; EGFR = epidermal growth factor receptor; *EGFR*ex20ins = *EGFR* exon 20 insertion; TKI = tyrosine kinase inhibitor; NOS = not otherwise specified

**Table 2:** Grade 2 or Greater Adverse Events Possibly, Probably, Definitely Related to Study Drug by CTCAE 4 Criteria

Adverse Event	DL0,^a^ *n* (%)				DL −1 (minus),^b^ *n* (%)			
	Course 1		Subsequent Courses		Course 1		Subsequent Courses	
	Grade 2	Grade 3	Grade 2	Grade 3	Grade 2	Grade 3	Grade 2	Grade 3
Abdominal pain	—	—	—	—	—	—	1 (13)	—
Activated partial thromboplastin time prolonged	1 (33)	—	—	—	—	—	—	—
Blood bilirubin increased	—	—	—	—	1 (13)	—	—	—
Colitis	1 (33)	—	1 (33)	—	—	—	—	—
Diarrhea	1(33)	1 (33)	—	—	2 (25)	2 (25)	1 (13)	3 (38)
Dyspepsia	—	—	—	—	—	—	1 (13)	—
Fatigue		—	—	—	1 (13)	—	1 (13)	—
Gastric hemorrhage	1 (33)	—	—	—	—	—	—	—
Generalized muscle weakness	1 (33)	—	—	—	—	—	—	—
Hypokalemia	—	—	—	—	—	—	1 (13)	—
Hypophosphatemia	—	1 (33)	1 (33)	—	—	—	—	—
Infusion related reaction	—	—	—	—	—	—	1 (13)	—
Lymphocyte count decreased	1 (33)	—	—	—	1 (13)	—	1 (13)	—
Nausea	—	—	—	—	—	—	1 (13)	—
Neutrophil count decreased	—	—	—	—	—	—	1 (13)	—
Pain in extremity	—	—	—	—	—	—	1 (13)	—
Rash acneiform	—	—	—	—	1 (13)	—	—	—
Rash maculopapular	—	1 (33)	—	—	—	—	—	—
Serum amylase increased	—	—	—	—	—	—	1 (13)	
Thrush	—	—	1 (33)	—	—	—	—	—

Abbreviations: CTCAE = Common Terminology Criteria for Adverse Events; DL0 = dose level 0; DL-1 = dose level −1; I.V. = intravenous.

a AT13387 (onalespib) 150 mg/m^2^ I.V. over 1 hour on days 1, 8, and 15; erlotinib 150 mg orally every morning (*n* = 3).

b AT13387 (onalespib) 120 mg/m^2^ I.V. over 1 hour on days 1, 8, and 15; erlotinib 150 mg orally every morning (*n* = 8).
