# Supplementary figures and images for: Erlotinib and Onalespib Lactate Focused on EGFR Exon 20 Insertion Non-Small Cell Lung Cancer (NSCLC): A California Cancer Consortium Phase I/II Trial (NCI 9878)
Source: Clin Lung Cancer. Author manuscript; Available in PMC 2022 Jun 28. (PMC9239707; doi:10.1016/j.cllc.2021.05.001)

## Slide 1
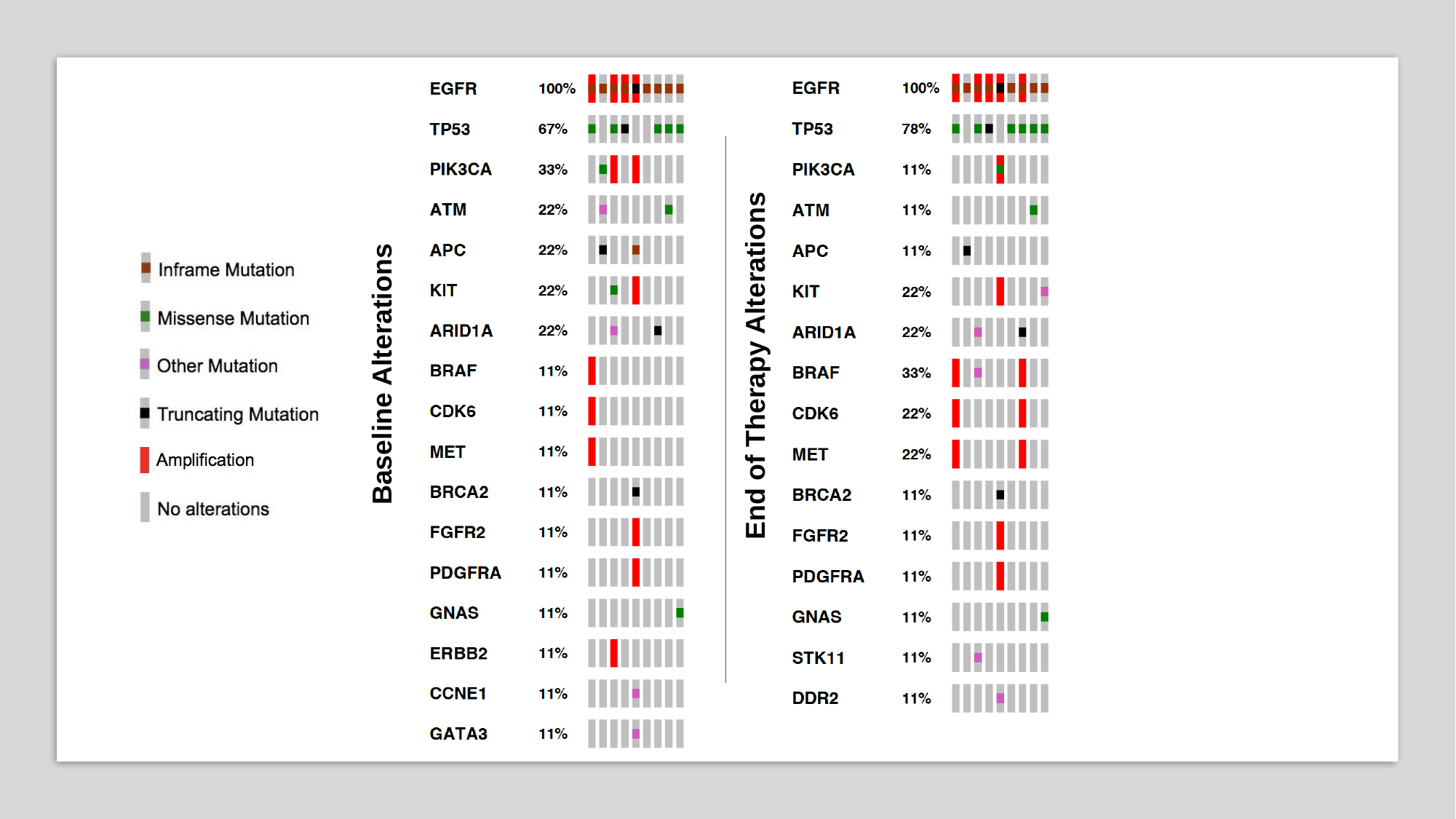

End of Therapy Alterations
Baseline Alterations

Supplement: 1 [file NIHMS1740680-supplement-1.pptx]
